# The Public’s Perception of Florence Nightingale’s Legacy in the Digital Media: A Critical Discourse Analysis

**DOI:** 10.3390/nursrep14030137

**Published:** 2024-07-24

**Authors:** Gianluca Conte, Arianna Magon, Maria Angela Palmeri, Giulia Paglione, Irene Baroni, Silvia Belloni, Miriam Angolani, Marco Alfredo Arcidiacono, Cristina Arrigoni, Alessandro Stievano, Rosario Caruso

**Affiliations:** 1 Health Professions Research and Development Unit, IRCCS Policlinico San Donato, 20097 San Donato Milanese, Italy; arianna.magon@grupposandonato.it (A.M.); giulia.paglione@grupposandonato.it (G.P.); irene.baroni@grupposandonato.it (I.B.); miriam.angolani@grupposandonato.it (M.A.); 2 Operating Theatre, IRCCS Istituto Ortopedico Galeazzi, 20157 Milan, Italy; mariaangela.palmeri@grupposandonato.it; 3 Department of Public Health, Experimental and Forensic Medicine, Section of Hygiene, University of Pavia, 27100 Pavia, Italy; silvia.belloni@unipv.it (S.B.); cristina.arrigoni@unipv.it (C.A.); 4 Medical Department, University Hospital of Parma, 43126 Parma, Italy; marcidiacono@ao.pr.it; 5 Department of Clinical and Experimental Medicine, University of Messina, 98100 Messina, Italy; alessandro.stievano@gmail.com; 6 Department of Biomedical Science for Health, University of Milan, 20133 Milan, Italy

**Keywords:** Nightingale, discourse, identity, image, nurse, nursing, podcasts, YouTube, digital, media

## Abstract

This study critically examines the public’s perception of Florence Nightingale’s legacy through a critical discourse analysis (CDA) of digital media, specifically podcasts and YouTube. Nightingale, who is often remembered as “The Lady with the Lamp”, holds a complex identity within modern narratives that is celebrated for her pioneering contributions to nursing and public health, even if there are some disagreements about her, given the colonialist setting that may have shaped some of her opinions and decisions. This research employed CDA to analyze 25 podcasts and 18 YouTube videos, which were systematically included according to a priori inclusion criteria. The study synthesized how these media products portray Nightingale and, by extension, shape public discourse about the nursing profession. The findings reveal five thematic representations of Nightingale: as a legendary figure, a modern feminist, a dedicated statistician, a pioneer in public health, and a pivotal STEM contributor. These portrayals challenge traditional nursing stereotypes by emphasizing Nightingale’s role as a rigorous scientist and reformer, suggesting broader perceptions of nurses that encompass leadership, analytical skills, and strategic thinking. The study supports the hypothesis that digital narratives significantly influence the public’s understanding and appreciation of nursing, advocating for a more nuanced professional identity that integrates traditional caregiving roles with critical and analytical capabilities.

## 1. Introduction

Florence Nightingale, often celebrated as the icon of modern nursing [1], remains a pivotal yet contested figure due to her significant contributions toward improving healthcare conditions during the Crimean War. In 1854, Nightingale and her team of 38 volunteer nurses implemented critical advancements in medical practices by addressing sanitary conditions and combating prevalent diseases like typhus, cholera, and dysentery, which were deadlier than battlefield injuries [2]. Her innovations in hygiene, diet, and the structure of healthcare facilities led to drastically lowered mortality rates and established foundational practices for infection control, shaping the landscape of modern nursing and healthcare [2,3].

Despite being lauded for her pioneering role, recent scholarly reviews present a more nuanced view of Nightingale [4]. They suggest that Nightingale was not merely a selfless caregiver but also a figure driven by personal ambition, at times manipulative, and even vindictive towards those who opposed her methods [5]. This complexity is evident in her extensive writings and work, which, while foundational in nursing education and practice, reflect the prejudices of her era, especially in the age of colonialism. Her views and policies, as contemporary scholarship notes, could be interpreted within the oppressive colonial system that perpetuated racial biases in the healthcare field [6,7].

Furthermore, the celebration of Nightingale as an icon is not universal. For example, countries in the Middle East reference historical figures such as Rufaida Al-Aslamia [8] and other significant Muslim women healers [9], showcasing different nursing legacies that are deeply rooted in their cultural contexts. This diversity challenges the assumption that Nightingale’s model of nursing is relevant across all global nursing communities. Also, recent academic discourse, such as the work described by Bates and Greenwood (2022), indicates that Nightingale’s commemorative culture has shifted dramatically since the 1850s, shaped by changing societal values around femininity, humanitarianism, Christianity, and nursing [10]. The rise of figures like Mary Seacole, particularly in the context of movements like Black Lives Matter, suggests a reevaluation of Nightingale’s position in nursing history, highlighting the need for a broader understanding of nursing icons across different cultures and epochs [11].

Despite the controversies, Nightingale’s intellectual rigor and extensive writings, including ‘Notes on Nursing’, remain widely used texts in nursing education in certain parts of the world, even though many have critiqued them [3,12]. This persistence of her work in educational settings highlights the duality of her legacy. While some in the nursing profession may view her primarily through her nurturing role, others acknowledge her as a complex figure who was both a scientist and a rigorous educator, capable of deep compassion but also marked by significant and critical flaws [2,3,4,5,13]. Nightingale seems to represent nursing for some, but not for all. Therefore, it is essential to consider what it means to evoke Nightingale as an icon for the nursing profession.

This internal viewpoint and debate within the nursing profession, however, often lack consideration of the general public’s perspective [3,14,15,16]. Understanding these varying perceptions could help bridge the gap between professional and public viewpoints, fostering a more comprehensive appreciation of Nightingale’s legacy by highlighting the differences between Florence Nightingale as a person and as an icon and its impact on the nursing field in both her positive and negative aspects.

In this scenario, modern digital platforms such as podcasts [17,18,19,20,21,22,23] and YouTube [24,25,26,27,28,29,30,31] play a pivotal role in constructing and contesting these kinds of public images. These digital platforms are not merely repositories of information but active fields where various portrayals of Nightingale and nursing, in general, are created and disseminated. For example, Kelly et al. (2012) highlighted how digital media, particularly YouTube, constructs nursing identities through stereotypes such as “the skilled knower and doer”, “the sexual plaything”, and “the witless incompetent”, providing critical insights into the public perception of nursing [32]. Thus, these digital narratives shape real public discourse, influencing nursing identities and perceptions [32].

This study hypothesizes that by analyzing the portrayals of Florence Nightingale on podcasts and YouTube, we are able to gain insights into how the public views nurses and nursing. Understanding these digital narratives could help clarify how nurses are perceived by the public, using Nightingale as a reference point. Such an analysis could inform strategies to counteract detrimental internal stereotypes and advocate for the profession’s advancement. Therefore, this study aims to describe Florence Nightingale’s identity as produced via podcasts and YouTube videos, ultimately providing a more updated understanding of both Florence Nightingale as an individual and a nurse’s public image.

## 2. Materials and Methods

This study employed critical discourse analysis (CDA) to describe, analyze, and synthesize the sources, examining how language reveals, conceals, reproduces, or challenges power ideologies and representations of self and others. CDA integrates various theoretical and methodological approaches from Applied Linguistics, Social Sciences, Cultural Studies, Anthropology, Ethnography, and Critical Pedagogy. This precise and rigorous method allows for analyzing everyday language texts, whether oral, written, or visual [33,34,35], making it an optimal approach for uncovering the significance of communication in social relationships.

### 2.1. Ethical Considerations

The team sought guidance from the institutional Ethics Committee associated with the last author regarding potential ethical concerns in presenting the findings from our analysis of video and audio content publicly available on the web. The committee indicated that using these video and audio clips as research data and disseminating the findings from our discourse analysis did not pose ethical concerns for the individuals featured in the videos as hosts. Consequently, the research did not undergo an ethical review.

### 2.2. Source Collection and Selection

The source search used digital media, primarily focusing on video contributions on YouTube and audio podcasts, excluding conventional databases. Digital media, which differ from traditional media like radio, print, and television due to their multimedia and interactive capabilities, were utilized to access web-based content. Notable characteristics of digital media include high-speed remote communication, vast storage potential, participatory features, and the absence of spatial-temporal boundaries [36]. The source selection was conducted from October 2023 to January 2024. The research strategy was systematic, independently managed by the research team, and followed different criteria for each media type.

### 2.3. Data Interpretation Strategy and Inclusion Criteria

Classical thematic saturation was not used to determine the number of videos and podcasts in this study. Instead, our focus was on interpreting the meanings embedded in the textual data of the video and audio items within specific social and historical contexts. This approach aligns with the methodology used by Kelly et al. (2012), which emphasized understanding public discourse through critical discourse analysis rather than seeking thematic saturation [32].

Initially, we reviewed and listened to a broad pool of sources to ensure comprehensive coverage. For YouTube videos, we identified the 500 most-viewed videos using the keyword “Florence Nightingale.” Similarly, we selected the top 100 relevant podcasts using the same keyword from public RSS-based databases and both iOS (Apple) and Android (Google) counterpart apps, ensuring systematic selection across platforms.

From this broad pool, we applied specific criteria to refine our selection to those sources most relevant to our research objectives. For YouTube videos, the selection criteria included at least 1000 views, a minimum duration of four minutes to ensure substantive content, exclusivity in English, and the exclusion of non-graphic content to avoid overlap with podcasts. For podcasts, the criteria included a minimum of 500 listens or downloads, a minimum duration of 15 min to ensure in-depth content, exclusivity in English, and availability on multiple platforms to ensure accessibility. Each source was evaluated for its relevance to the perception of Florence Nightingale, ensuring that only those with substantial content were included.

Following this, we investigated the backgrounds of the content creators to determine whether they were nurses, non-nurses, academics, or professionals from other fields. This review revealed that none of the content creators were from a nursing background or were academics in the field of nursing. Most were either historians, academics from other disciplines, or enthusiasts from the general public. At the end of the selection process, 25 podcasts (out of the initial 100 identified) and 18 videos (out of the initial 500 identified) were chosen for detailed analysis. These selected sources were then transcribed verbatim to obtain raw texts from video subtitles, accessible transcriptions for disabled users, or extracted using AI-driven audio-to-text conversion software (e.g., Otter.ai 3.31.0 and MacWhisper 7.9).

### 2.4. Data Analysis

One researcher independently reviewed all the transcriptions, confirming their inclusion in the study and establishing preliminary categories describing the various “identities” of Florence Nightingale evident in the texts. A second researcher independently analyzed the selected contents to ensure alignment with the predetermined categories. This methodological consistency facilitated an in-depth, rigorous analysis over three months, focusing on textual constructions of Nightingale’s identity through subject positions within the transcribed texts. More specifically, subject positions refer to socially constructed categories that are products of discourses and situated in context, culture, time, and place, which regulate what is possible or not possible to say, do, and feel about a particular subject [37].

Rigor in CDA was achieved by demonstrating the research’s validity, authenticity, and quality. Each element was analyzed to create a narrative synthesis describing each video or audio piece’s content, setting, and themes. Independent reviews by each researcher, followed by collective discussions, enhanced the rigor and credibility of the findings. The diverse professional backgrounds of the three researchers, all nurses with different specializations, added depth to the analysis, mirroring Nightingale’s multifaceted approach to her disciplines. This diverse expertise within the team, ranging from methodological scholarship to digital media proficiency and clinical coordination, ensured a comprehensive and authentic analysis. All the transcripts can be downloaded in the Supplementary File S1: Podcasts and YouTube Transcripts.

## 3. Results

The results are based on the digital narratives about Florence Nightingale, as portrayed in 25 podcasts and 18 YouTube videos that matched inclusion criteria among thousands of results. Nightingale is depicted in roles that highlight her historical and professional contributions rather than reductive or sensationalized images. This discrepancy underscores the different contexts and audiences addressed by digital media versus academic studies. Consequently, our analysis unearthed five prominent thematic representations of Florence Nightingale: (1) a legendary figure, (2) a modern feminist, (3) a dedicated statistician, (4) a pioneer in public health, and (5) a pivotal contributor to STEM fields. We provide a detailed account of these themes below.

### 3.1. The Legend

**Figure 1:**
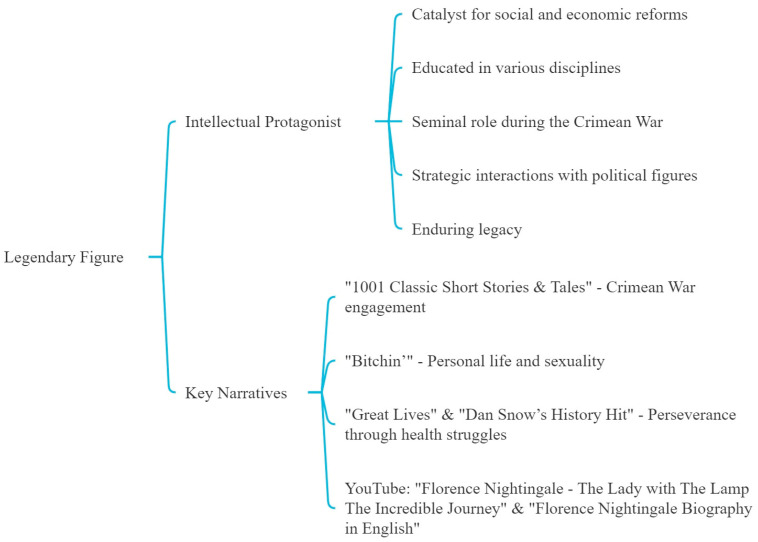
First theme: the legend.

Florence Nightingale emerged as a formidable and intellectual protagonist in narratives that portray her as a catalyst for social and economic reforms (Figure 1). She was educated extensively in various disciplines during her upbringing in a privileged English family, so her rigorous academic foundation was comparable to that of her male contemporaries. Our analysis highlights her seminal role during the Crimean War, strategic interactions with influential political figures, and enduring legacy that transcends generations.

In particular, the podcast “1001 Classic Short Stories & Tales” vividly depicts Nightingale’s engagement in the Crimean War. The narratives within “Bitchin’” delve into intimate aspects of her personal life, including speculations about her sexuality and personal relationships. “Great Lives” and “Dan Snow’s History Hit” reflect her perseverance through personal health struggles, emphasizing her indomitable spirit.

YouTube videos like “Florence Nightingale—The Lady with The Lamp The Incredible Journey” and “Florence Nightingale Biography in English” offer extensive biographical insights, focusing on her revolutionary approach to military hospital reforms and esteemed interactions with Queen Victoria. The video “Who Really Was Florence Nightingale?” discusses her significant contributions during the Crimean War and the broader implications of her work on modern nursing.

### 3.2. The Feminist Heroine

**Figure 2:**
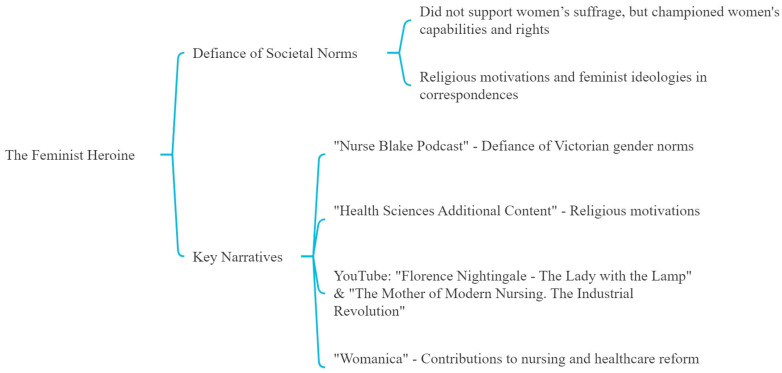
Second theme: the feminist heroine.

Nightingale is also depicted as a pioneer who vigorously challenged the societal norms of her era (Figure 2). Although she did not support the women’s suffrage movement, her actions and writings championed the capabilities and rights of women, positioning her as a complex figure within feminist narratives. The “Nurse Blake Podcast” discusses her defiance of Victorian gender norms, while “Health Sciences Additional Content” considers her religious motivations and feminist ideologies reflected in her extensive correspondences.

Visual narratives on YouTube such as “Florence Nightingale—The Lady with the Lamp” and “Florence Nightingale—The Mother of Modern Nursing. The Industrial Revolution” emphasizes her role in advocating for women’s rights, portraying her as an emblem of feminist resilience. The podcast “Womanica” also highlights Nightingale’s defiance against societal expectations and her significant contributions to nursing and healthcare reform.

### 3.3. The Statistician and Data Scientist

**Figure 3:**
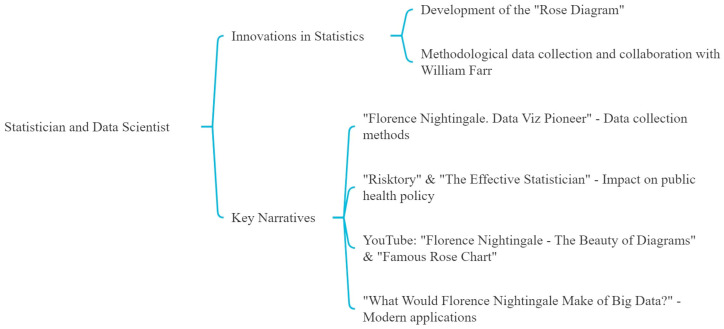
Third theme: the statistician and data scientist.

Nightingale’s innovative use of statistics and data visualization is central to several narratives, particularly her development of the “Rose Diagram” (Figure 3). The podcast “Florence Nightingale. Data Viz Pioneer” highlights her methodological data collection and her collaborative efforts with William Farr. “Risktory” and “The Effective Statistician” further explore her affinity for mathematics and the lasting impact of her statistical methodologies on public health policy and modern statistical practices.

Similarly, videos such as “Florence Nightingale—The Beauty of Diagrams” and “Florence Nightingale’s Famous Rose Chart” discuss her diagrams’ esthetic and functional significance, underscoring their relevance in contemporary data analysis across various sectors. The podcast “What Would Florence Nightingale Make of Big Data?” explores the modern applications of her statistical innovations in the context of big data.

### 3.4. The Public Health Innovator

**Figure 4:**
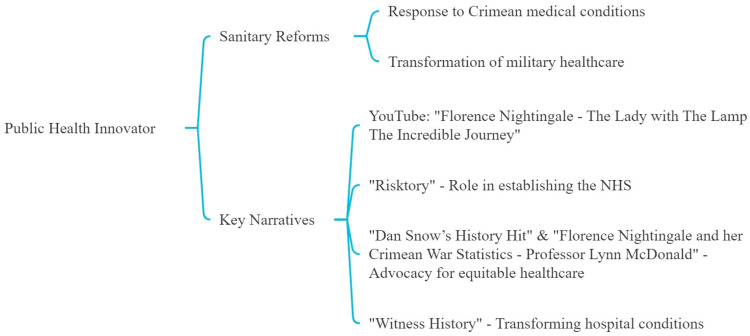
Fourth theme: the public health innovator.

Our analysis identifies Nightingale’s acute response to the dire medical conditions she encountered in Crimea, which prompted immediate and impactful sanitary reforms (Figure 4). Her pioneering efforts transformed military healthcare and profoundly influenced public health systems globally. “Florence Nightingale—The Lady with The Lamp The Incredible Journey” chronicles her extensive contributions to hospital reforms. At the same time, “Risktory” examines her foundational role in establishing the National Health Service (NHS).

Her advocacy for equitable healthcare and professionalization of nursing, highlighted in “Dan Snow’s History Hit” and the video “Florence Nightingale and her Crimean War Statistics—Professor Lynn McDonald”, exemplifies her holistic approach to healthcare reform. The podcast “Witness History” recounts her challenges and triumphs in transforming hospital conditions during the Crimean War and her subsequent influence on health policy.

### 3.5. The STEM Contributor

**Figure 5:**
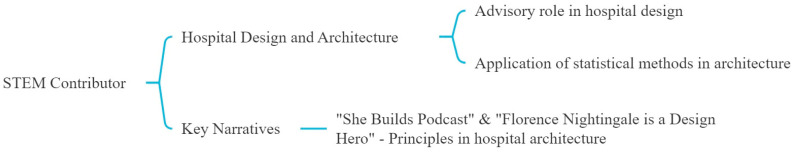
Fifth theme: the STEM contributor.

Nightingale’s advisory role in the design of hospitals and her application of statistical methods are illuminated. “She Builds Podcast” and “Florence Nightingale is a Design Hero” discuss her influential principles in hospital architecture (Figure 5), emphasizing how her ideas have shaped modern healthcare infrastructure. Her collaboration with architects and engineers showcases her interdisciplinary approach and lasting impact on hospital design.

## 4. Discussion

Exploring Florence Nightingale’s portrayal in digital media is pivotal for providing insights regarding nurses’ professional identity and understanding public perceptions of some aspects of nursing as reflected by the views on Florence Nightingale. Our study’s findings directly respond to our initial hypothesis that analyzing the portrayals of Florence Nightingale on podcasts and YouTube could give meaningful insights into how the public views nurses and nursing. The selection of media created outside of the nursing profession highlights a specific perspective in the portrayal of Nightingale. This distinction is essential as it brings to light the broader public’s view rather than an insider’s perspective. Furthermore, our findings diverge from those of Kelly and colleagues, who identified themes such as the “sexy nurse” stereotype [32]. In our study, we did not encounter such reductive portrayals. Instead, Nightingale is depicted in roles that emphasize her historical and professional contributions rather than sensationalized images. This discrepancy underscores the different contexts and audiences addressed by digital media versus academic studies, with the general public creating media aimed at providing inspiration to viewers and listeners who enjoy biographies and seek parallels in the modern world and society. These audiences seem to be more interested in a woman’s story than the profession’s one.

The analysis demonstrates that the general public portrays Florence Nightingale exclusively in a positive light, not only as a historical figure but also as a modern symbol of nursing whose legacy is reinterpreted through contemporary lenses. These digital narratives provide a spectrum of perceptions—from the traditional image of the nurturing caregiver to the less commonly recognized but equally significant roles of a public health innovator and a statistical genius. This diversity in representation challenges the limited image of nursing and showcases the profession’s complexity and depth.

Apart from the emerging positive views on the iconic figure of Florence Nightingale, it is important to acknowledge that some biographies depict her as ambitious, domineering, and manipulative, contrasting with her public image of selflessness and altruism [4,5]. Critiques have also highlighted her controversial spiritual beliefs and alleged unbalanced behavior. Additionally, modern critiques focus on her support for colonialism and inherent racism. Despite these negative aspects, our study found no negative descriptions from the general public, suggesting a disconnect between historical and professional critiques and contemporary perceptions. This highlights her enduring positive legacy in popular culture and underscores the importance of understanding these portrayals to inform strategies for advancing the nursing profession. Ultimately, Florence Nightingale as a ‘person’ and as an ‘icon’ are indeed different figures. Another significant turning point highlighted by our study is the realization that the enduring stereotypes of nursing—often centered around the notions of nurturing and compassion—can overshadow the analytical and leadership capabilities that are equally intrinsic to the profession. Even in architecture, as depicted in digital media, Nightingale’s legacy serves as a powerful reference point to counteract these stereotypes. By emphasizing her roles as a statistician, a reformer, and a healthcare strategist, the narratives provide a more comprehensive view that elevates the profession’s image. This broader perspective includes critical thinking, strategic planning, and complex problem-solving, thus showcasing the full spectrum of skills that nursing encompasses.

Florence Nightingale’s complex stance with regard to the women’s suffrage movement encapsulates the broader feminist debates of her time. Although she never fully embraced the movement, her life’s work—defying Victorian norms to forge a path in a male-dominated field—positions her as a proto-feminist figure. Today, these narratives resonate with ongoing discussions about gender roles within nursing, a field predominantly female yet often subject to gender-based leadership disparities. By dissecting these aspects, digital narratives invite a reevaluation of gender dynamics within the profession, promoting a view of nursing that advocates for leadership roles irrespective of gender, aligning with broader feminist ideals.

Our findings are instrumental in informing strategies for advancing the nursing profession. They suggest that nurses and the institutions that educate and support them use these broader portrayals to advocate for more substantial roles in healthcare policymaking, research, and leadership. Understanding how the public—shaped by these narratives—views nurses can help the profession advocate for roles that exceed traditional expectations, pushing for recognition in areas typically reserved for other healthcare professionals.

While this study provides valuable insights into the public portrayals of Florence Nightingale and their implications for nursing identity, several limitations warrant discussion. The study’s reliance on digital platforms such as YouTube and podcasts may have introduced a selection bias, as the content available on these platforms does not necessarily represent a comprehensive or balanced view of Florence Nightingale. Videos and podcasts with more views or higher popularity were more likely to be included, potentially skewing the analysis towards more mainstream or widely accepted narratives. The study focused exclusively on English-language content, which may not capture the full spectrum of Nightingale’s portrayal in non-English-speaking contexts. This limitation might have excluded diverse perspectives and cultural interpretations of her legacy, affecting the comprehensiveness of the analysis. While the study set minimum criteria for content inclusion (e.g., minimum views and duration), the quality and depth of the selected content varied. Some videos and podcasts may have provided more superficial or less rigorous analyses, affecting the overall depth and robustness of the findings. Moreover, CDA is inherently interpretative and subjective. The researchers’ backgrounds and perspectives may have influenced the analysis and interpretation of the content, potentially introducing bias. Although measures were taken to ensure rigor and consistency, the findings are ultimately shaped by the researchers’ interpretations. In addition, digital content is continually evolving, with new videos and podcasts being produced and existing ones being updated or removed. This dynamic nature means that the study’s findings represent a snapshot in time and may not reflect ongoing or future changes in how Florence Nightingale is portrayed. The study primarily analyzed the content without deeply exploring the audience’s reception or engagement with these narratives. Understanding how different audiences interpret and respond to these portrayals through sentiment analyses or other analytical approaches could offer additional insights into the impact of digital narratives on public perceptions of nursing.

Future research could address these limitations by incorporating a broader range of media sources, expanding the temporal and linguistic scope, and employing mixed methods to analyze both content and audience reception. Such approaches could further enrich our understanding of how historical figures like Florence Nightingale continue to shape contemporary professional identities and public perceptions in nursing and beyond.

## 5. Conclusions

This research affirms the hypothesis that examining the portrayals of Florence Nightingale in modern digital media could provide insights regarding public perceptions of nursing as reflected by the views on Florence Nightingale, offering a clearer and updated understanding of how the public views some aspects of nursing. These insights could be crucial for developing strategies to enhance the nursing profession’s public image and internal self-perception. While our study primarily analyzes how Nightingale is portrayed in digital media, it suggests the potential for these narratives to influence professional identities and public attitudes. It encourages nurses and nursing leaders to engage with and influence these narratives to ensure that they reflect the true diversity and complexity of the nursing profession. Furthermore, this research advocates for a redefined nurse identity that integrates the traditional values of care and empathy with the critical, analytical, and leadership skills essential for modern healthcare by highlighting Nightingale’s multifaceted legacy. In light of these findings, future research should focus on the direct impacts of these digital narratives on nursing education and policy, aiming to leverage Nightingale’s enduring legacy to foster a more nuanced understanding and appreciation of the nursing profession. To further contribute to consolidating the public’s internal and external picture of nursing, we support using the methodology used in this study to consolidate how the public perceives other scholars and key figures in modern nursing history on social media.

## Data Availability

Data are available in the Supplementary File.

## Supplementary Materials

The following supporting information can be downloaded at: https://www.mdpi.com/article/10.3390/nursrep14030137/s1, Supplementary File S1: Podcasts and YouTube Transcripts.

## Public Involvement Statement

There is no public involvement in any aspect of this research.

## Guidelines and Standards Statement

This manuscript was drafted against the Consolidated Criteria for Reporting Qualitative Research.
